# Comprehensive transcriptome profiling of urothelial cells following TNFα stimulation in an *in vitro* interstitial cystitis/bladder pain syndrome model

**DOI:** 10.3389/fimmu.2022.960667

**Published:** 2022-08-15

**Authors:** Tadeja Kuret, Dominika Peskar, Mateja Erdani Kreft, Andreja Erman, Peter Veranič

**Affiliations:** Institute of Cell Biology, Faculty of Medicine, University of Ljubljana, Ljubljana, Slovenia

**Keywords:** interstitial cystitis, bladder pain syndrome, urothelial cells, inflammation, RNA sequencing, transcriptomics, proinflammatory cytokines

## Abstract

Urothelial cells of the urinary bladder play a critical role in the development and progression of interstitial cystitis/bladder pain syndrome (IC/BPS), a chronic and debilitating inflammatory disease. Given the lack of data on the exact phenotype and function of urothelial cells in an inflammatory setting (as in IC/BPS), we performed the first in-depth characterization of these cells using RNA sequencing, qPCR, ELISA, Western blot, and immunofluorescence. After TNFα stimulation, urothelial cells in the *in vitro* model of IC/BPS showed marked upregulation of several proinflammatory mediators, such as SAA, C3, IFNGR1, IL1α, IL1β, IL8, IL23A, IL32, CXCL1, CXCL5, CXCL10, CXCL11, TNFAIPR, TNFRSF1B, and BIRC3, involved in processes and pathways of innate immunity, including granulocyte migration and chemotaxis, inflammatory response, and complement activation, as well as TLR-, NOD-like receptor- and NFkB-signaling pathways, suggesting their active role in shaping the local immune response of the bladder. Our study demonstrates that the TNFα-stimulated urothelial cells recapitulate key observations found in the bladders of patients with IC/BPS, underpinning their utility as a suitable *in vitro* model for understanding IC/BPS mechanisms and confirming the role of TNFα signaling as an important component of the associated pathology. The present study also identifies novel upregulated gene targets of TNFα in urothelial cells, including genes encoding the acute phase protein SAA, complement component C3, and the cytokine receptor IFNGR1, which could be exploited as therapeutic targets of IC/BPS. Altogether, our study provides a reference database of the phenotype of urothelial cells in an inflammatory environment that will not only increase our knowledge of their role in IC/BPS, but also advance our understanding of how urothelial cells shape tissue immunity in the bladder.

## 1 Introduction

Interstitial cystitis/bladder pain syndrome (IC/BPS) is a chronic disorder, generally characterized by discomfort or pain in the bladder and surrounding pelvic region, often accompanied by increased micturition, urgency, and nocturia (1). IC/BPS more commonly affects women with an estimated prevalence of 3–7% (2) and has severe and debilitating effects on quality of life (3). The diagnosis of IC/BPS is mostly based on exclusion criteria for other conditions, with no specific and reliable diagnostic biomarkers available to date (4, 5). Currently, there is no treatment that has been consistently successful in alleviating clinical symptoms and ensuring long-term efficacy in patients with IC/BPS (6).

The exact etiology and pathophysiology of IC/BPS still remain elusive, however, a substantial number of studies have suggested injury or dysfunction of the urinary bladder urothelium and continuous cycle of inflammation as key components (5, 7). The urothelium is a specialized type of epithelial tissue that lines the wall of the majority of the urinary tract and forms a blood-urine barrier that prevents the penetration of urine components into the underlying tissue and bloodstream (8, 9). Damage to the urothelial layer increases permeability, allows urinary solutes such as urea and potassium to permeate into the bladder wall, and leads to activation of an inflammatory response, as well as mast cell infiltration with increased production of several proinflammatory mediators, such as tumor necrosis factor α (TNFα), interleukin (IL)1β and IL8. These mediators sensitize afferent nerve endings, leading to increased release of neuropeptides that promote mast cell degranulation and further contribute to the inflammatory process (10–12).

TNFα is a proinflammatory cytokine that plays an important role in the pathogenesis of IC/BPS and acts as a mediator of the urothelial response to mast cell secretion products (13). Its expression has been found to be increased in bladder urothelium (14, 15), and serum (16) of IC/BPS patients compared to healthy controls. Furthermore, TNFα signaling pathway has been determined as significantly enriched in the bladders of IC/BPS patients, specifically in those with Hunner’s lesions (17). It is therefore not surprising that published *in vitro* models of bladder urothelial cells frequently use TNFα to mimic an inflammatory milieu found in IC/BPS (18–23). However, the exact effects of TNFα on global gene expression and alterations of cellular processes in urothelial cells are still not yet entirely known. Hence, we here determined the overall effect of TNFα on the transcriptomic profile of urothelial cells using RNA sequencing (RNA seq) and validated our result using qPCR, ELISA, Western blots, and immunofluorescence. The concentration of TNFα (20 ng/ml) that was used to stimulate urothelial cells in the current study was selected based on previously published *in vitro* studies (18–23), viability assays and the ability to elicit a maximum measurable inflammatory response with the methods used that reached statistical significance. The ultimate goal of the *in vitro* model was to recapitulate the most important pathological processes and pathways, found enriched in the bladders of IC/BPS patients, and identify new potential therapeutic targets. As normal human bladder urothelial cells are difficult to obtain for ethical reasons, while cell lines are widely accessible, our *in vitro* model consisted of the human non-invasive bladder cancer urothelial cell line RT4. In contrast to the more commonly used muscle-invasive bladder cancer urothelial cell line T24 in previously published *in vitro* IC/BPS models (18, 20, 21, 23), RT4 cells isolated from grade I transitional cell carcinoma better reflect normal urothelial cells, especially with respect to differentiation markers (24, 25), as well as growth and migration characteristics (26). However, to increase the credibility of our results, we also confirmed our main findings from RT4 cells in normal porcine bladder urothelial (NPU) cells, which are more accessible and share most structural, molecular, and physiological features with normal human bladder urothelial cells (27–29). Overall, our study provides a reference database and the foundation for future studies to explore potential diagnostic biomarkers and therapeutic targets for IC/PBS.

## 2 Methods

### 2.1 *In vitro* models of interstitial cystitis/bladder pain syndrome

#### 2.1.1 TNFα-treated RT4 cell line

Human non-invasive cancer urothelial cells, RT4, isolated from transitional cell papilloma (HTB-2, ATCC, Manassas, VA) were grown in 75 cm^2^ cell culture flasks in basal media consisting of equal parts of advanced Dulbecco’s modified Eagle’s medium (A-DMEM) (Gibco, Thermo Fisher Scientific, Waltham, MA, USA) and F12 (HAM) (Sigma Aldrich, St. Louis, MO, USA), supplemented with 5% fetal bovine serum (FBS), and 4 mM GlutaMAX (both Gibco, Thermo Fisher Scientific, USA). Cells repeatedly tested negative for mycoplasma infection using MycoAlert mycoplasma detection kit (Lonza, Basel, Switzerland). For the experiments, RT4 cells were seeded in 6-well plates with (for IF) or without (for RNA and protein extraction and measurement of protein levels in supernatants) coverslips or 96-well plates (for viability assay) at a seeding density of 5×10^4^ cells/cm^2^ and grown until reaching 80-90% confluency (approximately 3-4 days). To mimic a proinflammatory environment, cells were treated with 20 ng/ml human recombinant TNFα (Cayman Chemicals, Ann Arbor, MI, USA) for 24 h in serum-free basal media, as previously described (18–21). Untreated cells grown in serum-free basal media served as controls.

#### 2.1.2 TNFα-treated normal porcine urothelial cells

NPU cells were established from porcine urinary bladders (n=5), obtained independently from a local abattoir as described in (27–29). The use of porcine urinary bladders for preparation of primary urothelial cells was approved by the Administration for Food Safety, Veterinary Sector and Plant Protection of the Slovenian Ministry of Agriculture and Forestry in compliance with the Animal Health Protection Act and the Instructions for Granting Permits for Animal Experimentation for Scientific Purposes. The NPU cell cultures were grown in 75 cm^2^ cell culture flasks in basal UroM medium, consisting of equal parts of MCDB153 medium (Sigma-Aldrich, USA) and A-DMEM (Thermo Fisher Scientific, USA), supplemented with 2.5% FBS (Gibco, Thermo Fisher Scientific, USA), 0.1 mM phosphoethanolamine, 15 µg/mL adenine, 0.5 µg/mL hydrocortisone, 5 µg/mL insulin (all Sigma-Aldrich, USA), and 4 mM glutamax (Gibco, Thermo Fisher Scientific, USA). For experiments, NPU cells were seeded in 6-well plates with (for IF) or without (for RNA and protein extraction and measurement of protein levels in supernatants) coverslips or 96-well plates (for viability test) at a seeding density of 1×10^5^ cells/cm^2^ and grown until reaching 80-90% confluency (approximately 4-5 days). To mimic a proinflammatory environment, cells were treated with 20 ng/ml porcine recombinant TNFα (R&D Systems, Minneapolis, MN, USA) for 48 h in serum-free basal media. Untreated cells, grown in serum-free basal media served as controls. All cell cultures were maintained at 37°C in a humidified atmosphere at 5% CO2.

### 2.2 Viability assay

For viability assay, RT4 or NPU cells were seeded in 96-well plates and grown until reaching 80-90% confluency. Subsequently, cells were treated with increasing concentrations of TNFα (0, 2, 10, 20, 50, 100 ng/ml) in serum-free basal media for 24 h (RT4 cells) or 48 h (NPU cells). Cell viability was determined using CellTiter-Glo^®^ Luminescent Cell Viability Assay (Promega, Madison, WI, USA) following manufacturer’s instructions. Luminescent signal proportional to the amount of ATP present was subsequently measured using a microplate reader (Safire; Tecan, Mannedorf, Switzerland). Viability assay was performed in triplicates in 3 independent experiments (RT4) or 3 biological replicates (NPU). The results were expressed as percentage of luminescence signal intensity of untreated controls (set to 100).

### 2.3 RNA isolation

For RNA seq and qPCR, total RNA was isolated from RT4 (n=8 independent experiments) and NPU cells (n=5 biological replicates), grown on 6-well plates, using Quick-RNA Microprep Kit (Zymo Research, Irvine, CA, USA), according to manufacturer’s instructions with on column genomic DNA digestion. The concentration and purity of isolated RNA were assessed with Qubit RNA Broad Range Assay Kit on Qubit Flex Fluorimeter (both Invitrogen, Thermo Fisher Scientific, Waltham, MA, USA) and NanoDropTM 1000 (Thermo Fisher Scientific), respectively.

### 2.4 RNA sequencing

RNA seq was performed in RT4 cells (n=3 independent experiments) by Novogene Co., Ltd. (Beijing, China). Prior to library preparation, the integrity of RNA, isolated from RT4 cells was assessed using the RNA Nano 6000 Assay Kit and the Bioanalyzer 2100 system (Agilent Technologies, Santa Clara, CA, USA). A total amount of 0.5 µg RNA per sample was used as an input material for the RNA sample preparations. RNA included in the analysis had a RNA integrity number (RIN) ≥ 7. Sequencing libraries were generated using NEBNext^®^ UltraTM RNA Library Prep Kit for Illumina (NEB, Ipswich, MA, USA) following manufacturer’s recommendations. Then PCR was performed with Phusion High-Fidelity DNA polymerase, Universal PCR primers and Index (X) Primer. PCR products were purified (AMPure XP system) and library quality was assessed on the Agilent Bioanalyzer 2100 system. The clustering of the index-coded samples was performed on a cBot Cluster Generation System using TruSeq PE Cluster Kit v3-cBot-HS (Illumina, San Diego, CA, USA) according to the manufacturer’s instructions. After cluster generation, the library preparations were sequenced to a read depth of 26 million reads/sample, on an Illumina Novaseq 6000 platform, generating paired-end 150 bp reads fastq files. Original raw data can be found in the NCBI Gene Expression Omnibus database with the accession number GSE202576.

Raw data (raw reads) of fastq format were firstly processed through Novogene Co., Ltd. in-house perl scripts. In this step, clean data (clean reads) were obtained by removing reads containing adapters, reads containing ploy-N and low quality reads from raw data. At the same time, Q20, Q30 and GC content of the clean data were calculated. High quality reads were aligned to human (Ensembl GRCh38.p13) reference genome using Hisat2 (v2.0.5).

FeatureCounts (v1.5.0-p3) was used to count the reads numbers mapped to each gene. The fragments per kilobase of transcript sequence per millions base pairs sequenced (FPKM) of each gene was calculated based on the length of the gene and reads count mapped to this gene. RNA seq reads were of good quality with ~ 93% reads mapping uniquely to the reference genome.

### 2.5 Gene enrichment and pathway analysis of differentially expressed genes

Differential expression analysis of two groups (TNFα-treated vs. control; 3 replicates per group) was performed by Novogene Inc., using DESeq2 R package (1.20.0). The p value was calculated using the negative binomial distribution and the resulting p values were adjusted using the Benjamini-Hochberg’s approach for controlling the false discovery rate (FDR). The clusterProfiler (v. 3.8.1) software was used for enrichment analysis of differentially expressed genes, including Gene Ontology (GO) enrichment analysis and Kyoto Encyclopedia of Genes and Genomes (KEGG) database. Pathways with adjusted p values less than 0.05 were considered as significantly enriched by differentially expressed genes. The protein-protein interaction network was constructed for differentially expressed genes by searching STRING protein interaction database.

### 2.6 Reverse transcription and qPCR

The expression profile of selected genes was confirmed using qPCR performed on RNA samples isolated from RT4 and NPU cells. For RT4 cells, three independent experiments employed in RNA seq and five additional experiments (controls vs TNFα-treated cells; n=8 in total) were used. For NPU cells, RNA was isolated from 5 biological replicates (TNFα-treated and controls). Reverse transcription of 1 ug of total RNA/sample was performed with Promega Reverse Transcription System Kit (Promega, Madison, WI, USA) following manufacturer’s instructions. qPCR analysis was performed in triplicates on LightCycler^®^ 480 PCR System in LightCycler^®^ 480 Multiwell Plates 384 (both Roche, Basel, Switzerland), using self-designed primers (Integrated DNA Technologies, Coralville, IA, USA) and 5x HOT FIREPol EvaGreen qPCR Mix Plus (Solis BioDyne, Tartu, Estonia). Sequences of primers used for qPCR are listed in **Supplementary Table S1**. Expression of GAPDH or ACTB were used as endogenous control to normalize the data of RT4 and NPU cells, respectively. Data were analyzed with the comparative 2^-ΔΔCt method and presented as fold change of TNFα-treated cells vs untreated controls (set to 1).

### 2.7 Immunofluorescence

For F-actin and IFNGR1 labeling, both RT4 and NPU cells were seeded in 6–well plates with coverslips. After 24 h (RT4) or 48 h (NPU) of TNFα-treatment, cells were fixed in 4% paraformaldehyde in PBS. After fixation, the samples were washed in PBS and blocked with 1% BSA/PBS, at room temperature for 1 h. The samples were then incubated with phalloidin conjugated with FITC (diluted 1:5 in PBS, P6282; Sigma-Aldrich, USA) for 1 h, at RT, and washed in PBS. For immunolabeling with anti-IFNGR1, the samples were permeabilized for 10 min with 0.1% TritonX-100 (Sigma Aldrich, St. Louis, MO, USA) prior to blocking in 1% BSA/PBS, at RT, 1 h. Subsequently, samples were incubated with anti-IFNGR1 rabbit monoclonal antibody (ab134070, Abcam, Cambridge, United Kingdom), diluted 1:100 in 1% BSA/PBS overnight at 4°C. For negative control, the primary antibodies were omitted and samples were incubated in 1% BSA in PBS at 4°C overnight. After washing in PBS, secondary goat anti-rabbit antibodies (Alexa Fluor 488; Invitrogen, Molecular Probes, Thermo Fisher Scientific), diluted 1:400 in 1% BSA/BPS were added for 2 h, at RT. After washing in PBS, the samples were mounted in Vectashield mounting medium with 4′,6-diamidino-2-phenylindole (DAPI) (Vector Laboratories, Burlingame, CA, USA) for DNA labeling. The samples were analyzed with a fluorescence microscope AxioImager.Z1 equipped with ApoTome (Carl Zeiss MicroImaging GmbH, München, Germany). Immunofluorescence images shown here are representative of 3 independent experiments (RT4) and 3 biological replicates (NPU).

### 2.8 Enzyme-linked immunoassays

The supernatants of RT4 and NPU cells, seeded in 6-well plates and treated with/without TNFα for 24 h or 48 h, were collected, centrifuged (200 × g, 5 min, RT) and stored at -80°C until analysis. Enzyme-linked immunoassays (ELISA) were performed using commercial ELISA kits in duplicates, according to manufacturer’s instructions. Absorbance at 450 nm with a reference wavelength set at 570 nm was measured on a microplate reader (Safire; Tecan, Mannedorf, Switzerland). The following ELISA kits were used in the present study: human IL8 ELISA MAX™ Deluxe Set, human IL1A MAX™ Deluxe Set, human IL32 MAX™ Deluxe Set, human CXCL10 ELISA MAX™ Deluxe Set (all Bio Legend, San Diego, CA, USA), human CXCL1 Duo Set Kit (R&D Systems, USA), human complement C3 ELISA Kit (Novus Biologicals, Englewood, CO, USA), and porcine IL8/CXCL8 Quantikine ELISA Kit (R&D Systems, USA).

### 2.9 Western blots

For western blots, RT4 and NPU cells were seeded in 6-well plates and treated with/without TNFα for 24 or 48 h, respectively. After the treatment, the RT4 and NPU cells were collected and lysed in ice-cold RIPA lysis buffer (Merck, Kenilworth, NJ, USA), containing a cocktail of protease and phosphatase inhibitors (Thermo Fisher Scientific, USA). Total protein levels were quantified using the Pierce BCA Protein Assay Kit (Thermo Fisher Scientific, USA). Equivalent concentrations of protein (20 µg/lane) were separated using 4–20% Novex WedgeWell Tris-Glycine Gels (Invitrogen, Carlsbad, CA, USA) and then transferred onto a nitrocellulose membrane (Sigma-Aldrich, USA). The membranes were blocked in blocking buffer consisting of 5% skim milk in 0.1% Tris Buffered saline/Tween 20 (TBS-T) for 2 h at RT and incubated overnight at 4°C with primary antibodies against IFNGR1 (diluted 1:1000 in blocking buffer; ab134070, Abcam, UK) and anti-α-tubulin (diluted 1:2000 in blocking buffer; T6199, Sigma-Aldrich, USA). The next day, the membranes were washed with TBS-T and immediately incubated for 1 h at RT with secondary antibodies conjugated with horseradish peroxidase (diluted 1:1000 in blocking buffer, A6154, Sigma-Aldrich, USA). Visualization of the protein bands was performed using the SuperSignal West Pico Chemiluminescent Substrate (Thermo Fisher Scientific, USA), and the iBright FL1500 imaging system (Thermo Fisher Scientific, USA). iBright Firmware 1.7. (Thermo Fisher Scientific, USA) was used to perform the densitometric analysis, normalized to the expression of α-tubulin, used as a loading control. Western blot analyses shown here are representative of 5 independent experiments (RT4) and 4 biological replicates (NPU).

### 2.10 Statistical analysis

Statistical analysis was performed using Graph Pad Prism software 8.01 (Graphpad Software Inc., San Diego, CA, USA). The normality of data distribution was investigated by the Shapiro-Wilk test. Due to the normal distribution of the data, summary statistics are expressed as means and standard deviations (SD) unless otherwise stated. One-sample t-test (normal distribution) was used to compare the x-fold change of treated versus control (set to 1) groups. Statistical differences between two groups were calculated using unpaired t-test with (unequal variances) or without (equal variances) Welch’s correction for variables with normal data distribution. All tests were two-tailed and p values of <0.05 were regarded as statistically significant.

## 3 Results

### 3.1 Initial validation of the *in vitro* IC/BPS model of RT4 urothelial cells

**Figure 1:**
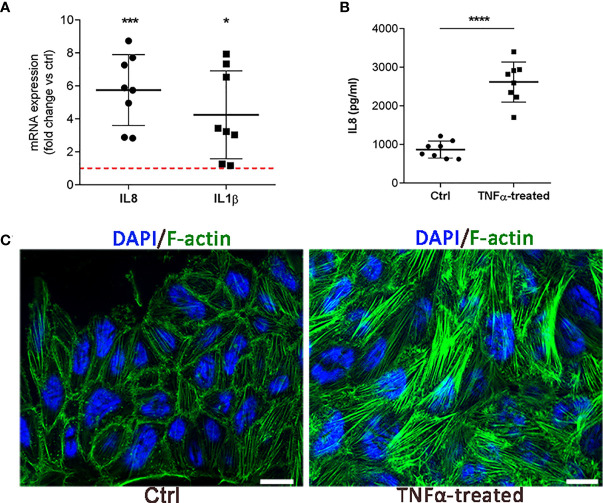
Initial validation of the *in vitro* IC/BPS model on RT4 cells. **(A)** mRNA expression of IL1β and IL8 in TNFα-treated cells compared to untreated cells (ctrl, red dotted line). Shown is mean ± SD fold change of TNFα-treated cells (n=8) vs. ctrl (n=8; set to 1 indicated with red dotted line). **(B)** Protein levels of IL8 in the supernatants of TNFα-treated cells and ctrl (n=8 each). Shown are mean ± SD protein levels for each group. **(C)** Representative images of 3 independent experiments showing F-actin (green) and DAPI (blue) of ctrl and TNFα-treated cells. Z-stack images were taken at the 63 x magnification at the same starting position and with the same distance (0.24 μm) between optical sections in ctrl and TNFα-treated samples. Scale bars: 10 µm. *p<0.05; ***p<0.001; ****p<0.0001.

Based on the initial cell viability assay, stimulation with 20 ng/ml TNFα for 24 h was selected as optimal to create an inflammatory environment in the *in vitro* model of RT4 urothelial cells (**Supplementary Figure 1**). Initial assessment of the *in vitro* model included measurement of gene expression of two inflammatory markers (*IL1β* and *IL8*) known to be upregulated in urothelial cells after TNFα stimulation (18, 20) and assessment of cell morphologic changes. In addition to increased mRNA expression of *IL1β* and *IL8* (**Figure 1A**), we also detected increased protein levels of IL8 (**Figure 1B**) in the supernatants of RT4 cells after 24 h of TNFα treatment. As expected, TNFα-treated cells enhanced formation of long and thick actin stress fibers that were randomly distributed throughout the cell, whereas in untreated cells phalloidin staining was observed mainly in the cell cortex (**Figure 1C**). This finding showing actin remodeling and cytoskeletal rearrangement is commonly observed in TNFα-treated endothelial cells (30, 31). The upregulated expression of two major proinflammatory cytokines together with enhanced formation of stress fibers indicated that RT4 urothelial cells were in an inflammatory state induced by TNFα.

### 3.2 The transcriptome of TNFα-treated RT4 urothelial cells is enriched in innate immunity signaling pathways

**Figure 2:**
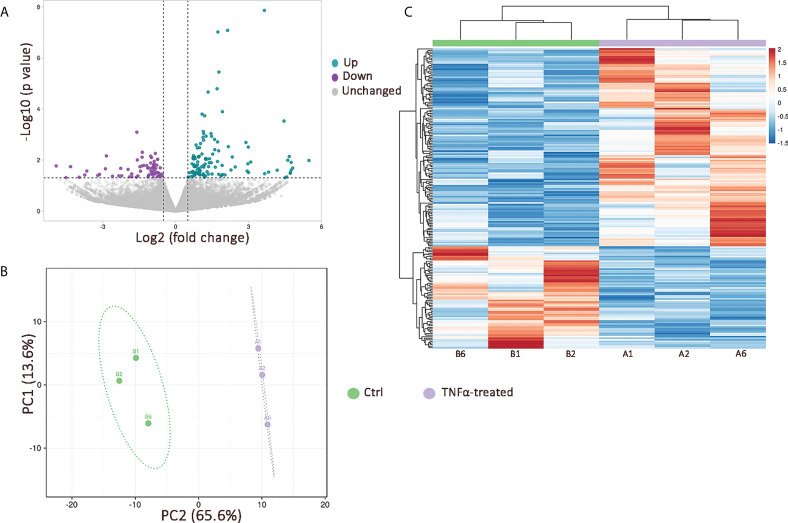
The effects of TNFα stimulation on transcriptional profile of RT4 urothelial cells *in vitro*. **(A)** Volcano plot showing 127 upregulated (green dots) and 72 downregulated (purple dots) genes in TNFα-treated cells compared to controls (p value ≤ 0.05; log2 foldchange ≥ 0.5); **(B)** Principal component analysis based on 199 DEGs with principal components 1 and 2 (PC1 and PC2) explaining 13.6% and 65.4% of the total variance, respectively. Unit variance scaling was applied to rows and singular value decomposition (SVD) with imputation was used to calculate principal components. Prediction ellipses are such that with probability 0.95, a new observation from the same group will fall inside the ellipse; **(C)** Heatmap with unsupervised hierarchical clustering based on 199 DEGs showing a clear distinction between TNFα-treated (purple; n=3 independent experiments: A1, A2, A6) and control (green; n=3 independent experiments: B1, B2, B6) cells. Both rows and columns are clustered using Euclidean distance and average linkage.

To comprehensively characterize the transcriptome of RT4 urothelial cells exposed to an inflammatory environment, we performed RNA seq in TNFα-treated vs. untreated cells (n=3 independent experiments). Differential expression analysis identified 199 differentially expressed genes (DEG; p ≤ 0.05), of which 127 were upregulated and 72 were downregulated (**Figure 2A**). Unsupervised principal component (PC) analysis, based on all 199 DEGs showed a clear separation between TNFα-treated and untreated cells, with PC1 and PC2 explaining 13.6 and 65.6% of the total variability, respectively (**Figure 2B**). This analysis showed that the main variance between independent experiments was TNFα stimulation (PC1). DEGs were further visualized by unsupervised hierarchical clustering, which indicated clustering of samples according to the degree of similarities in gene expression patterns, with a clear distinction between TNFα-treated and ctrl cells (**Figure 2C**).

**Figure 3:**
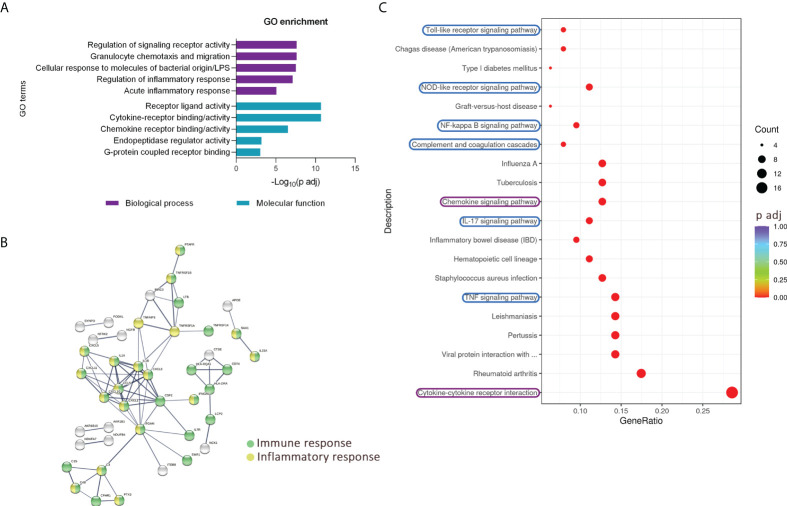
Upregulated transcripts in TNFα-treated RT4 urothelial cells are significantly enriched in pathways of innate immunity. **(A)** GO enrichment analysis based on upregulated genes showing five top enriched biological processes (purple) and molecular functions (blue) ranked by the lowest adjusted p value. **(B)** STRING analysis shows multiple known and predicted interactions, including co-expression among the TNFα-upregulated genes and their putative proteins, involved mainly in immune (green) and inflammatory (yellow) responses. Only protein interactions of high confidence are shown. **(C)** Dot plot of KEGG enriched pathways. The 20 KEGG enriched pathways with the lowest adjusted p value (based on significantly upregulated transcripts in TNFα-treated cells) showing several enriched immune response pathways, especially signaling cascades of innate immunity (highlighted in blue), as well as chemokine signaling pathway and cytokine-cytokine receptor interaction (highlighted in purple). The size of the dots represents the number of genes, while the color of the dots represents the adjusted p value in the significantly upregulated gene list associated with the individual KEGG pathway.

To better understand which pathways and biological processes are activated in urothelial cells during inflammation, we performed KEGG and GO enrichment analysis based on the upregulated transcripts in TNFα-stimulated cells. The majority of the upregulated transcripts were enriched in processes and signaling pathways involved in the innate immune response. The top five enriched biological processes identified by GO were related to regulation of signaling receptor activity, granulocyte chemotaxis and migration, cellular response to molecules of bacterial origin and lipopolysaccharide (LPS), regulation of inflammatory response, and acute inflammatory response. The top five enriched GO molecular functions included receptor ligand activity, cytokine receptor binding/activity, chemokine receptor binding/activity, endopeptidase regulatory activity, and G protein-coupled receptor binding (**Figure 3A**). Several interactions, including coexpression, are evident between the upregulated genes and their putative proteins involved in the immune and inflammatory response, as shown by STRING analysis (**Figure 3B**). The enriched KEGG signaling pathways in the network yielded similar results, as several signaling pathways involved in innate immunity, such as Toll-like and NOD-like receptor-signaling pathways, NFkB-, TNF-, and IL17-signaling pathways, and complement and coagulation cascades, were identified. In addition, cytokine-receptor interactions and chemokine signaling pathways were also among the twenty most enriched KEGG pathways (**Figure 3C**).

### 3.3 TNFα-treated RT4 urothelial cells upregulate acute inflammatory response genes and produce higher levels of complement component C3

**Figure 4:**
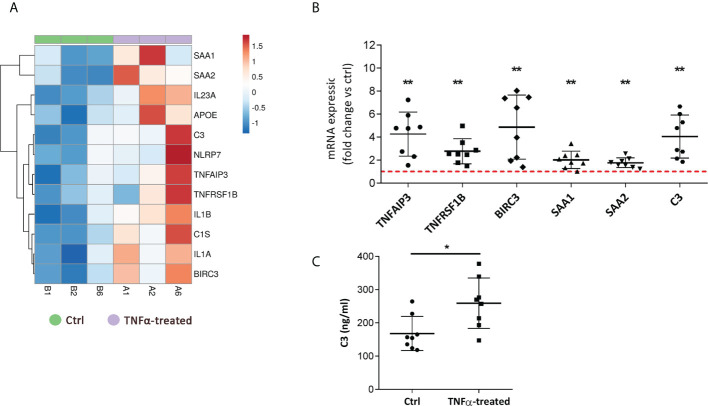
TNFα-treated RT4 urothelial cells upregulate the expression of genes involved in acute inflammatory response and complement and coagulation cascade. **(A)** Heatmap showing upregulation of transcripts (RNA seq), enriched in regulation of inflammatory and acute inflammatory response, in TNFα-treated urothelial cells (purple, n=3 independent experiments: A1, A2, A6) vs ctrl cells (green, n=3 independent experiments: B1, B2, B6). Rows are clustered using Euclidean distance and average linkage. Lower expression is indicated with blue, higher expression is indicated with red. **(B)** qPCR validation of selected genes of acute inflammatory response confirming their upregulation in TNFα-treated cells. Shown is mean ± SD fold change expression of target genes in TNFα-treated cells vs ctrl (set to 1; dotted red line), determined in 8 independent experiments. **(C)** Confirmatory analysis showing significantly higher protein levels of complement component C3 released in supernatants of TNFα-treated cells compared to controls (n=8 in each experimental condition). Shown are mean ± SD for control and TNFα-treated groups. *p<0.05; **p<0.01.

We next validated the RNA seq data by performing independent qPCR analysis of selected upregulated genes involved in the regulation of inflammatory response (**Figure 4A**), including *TNFAIP3, TNFRSF1B, BIRC3, SAA1, SAA2*, and *C3*, a component also implicated in the complement and coagulation cascade. These two processes have been previously already identified as enriched in the bladders of patients with IC/BPS (17, 32), but not yet in *in vitro* models. Our validation qPCR analysis showed significant upregulation of all measured transcripts in TNFα-treated cells compared to controls (**Figure 4B**). Furthermore, the protein levels of C3 were significantly increased in supernatants of TNFα-treated cells vs controls (**Figure 4C**).

### 3.4 TNFα-treated RT4 urothelial cells are primed for increased production of proinflammatory cytokines and chemokines

**Figure 5:**
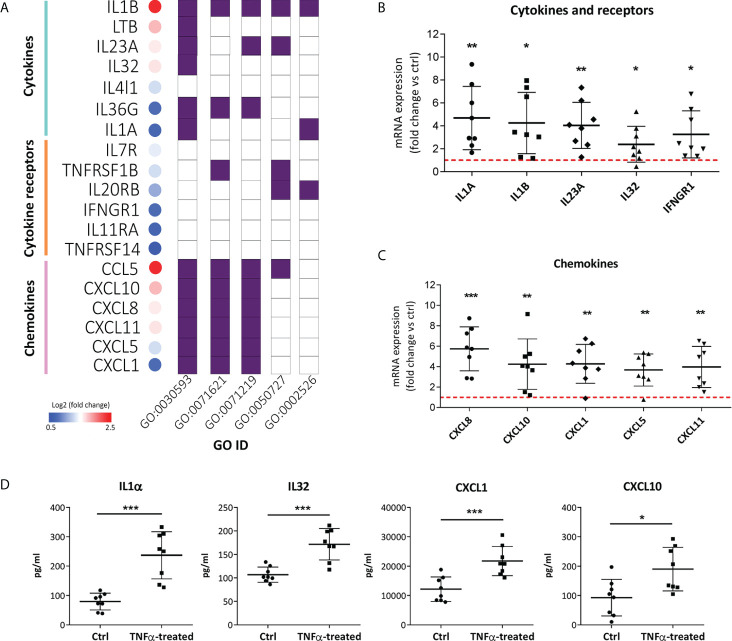
TNFα-treated RT4 urothelial cells are primed for enhanced proinflammatory cytokine and chemokine production. **(A)** Significantly upregulated mRNA expression of cytokines, cytokine receptors and chemokines in TNFα-treated samples vs controls with log2 fold change ranging from 0.5 (blue) to 2.5 (red), as determined by RNA seq. The majority of upregulated cytokines, cytokine-receptors and chemokines were significantly enriched in the following GO biological processes: regulation of signaling receptor activity (GO:0030593), granulocyte chemotaxis/migration (GO:0071621), cellular response to molecules of bacterial origin/LPS (GO:0071219), regulation of inflammatory response (GO:0050727), and acute inflammatory response (GO:0002526). B, C) qPCR confirmation of significantly upregulated mRNA expression of cytokines, cytokine receptors **(B)** and chemokines **(C)** in TNFα-treated urothelial cells vs controls. Shown is mean ± SD fold change expression of target genes in TNF-α treated cells vs untreated cells (ctrl; set to 1; red dotted line), determined in 8 independent experiments. **(D)** Confirmatory analysis showing significantly higher protein levels of IL1α, IL32, CXCL1 and CXCL10 released in supernatants of TNFα-treated cells compared to controls (n=8 in each experimental condition). Shown are mean ± SD for control and TNFα-treated groups. *p<0.05; **p<0.01; ***p<0.001.

In addition to innate immunity pathways, KEGG enrichment analysis showed that chemokine signaling and cytokine-cytokine receptor interactions were among the 20 most enriched processes in the TNFα-regulated gene network (**Figure 3C**). The same was observed in the GO enrichment analysis in terms of molecular function, as receptor-ligand activity, cytokine-receptor binding/activity, and chemokine-receptor binding/activity were among the five most enriched functions in our gene set of interest (**Figure 3A**). While increased expression of IL8 and IL1β has been reported in previous studies (11, 13), this is the first report of increased mRNA expression of *IL1α, IL23A, IL32, IFNGR1, CXCL1, CXCL10, CXCL11*, and *CXCL5* in RT4 urothelial cells after TNFα stimulation (**Figure 5A**). To confirm our results from RNA seq, independent qPCR analysis was performed, which showed significant upregulation of all the aforementioned proinflammatory cytokines and chemokines in TNFα-treated cells compared with untreated controls (**Figures 5B, C**). In addition, protein levels of IL1α, IL32, CXCL1, and CXCL10 were significantly increased in the supernatant of TNFα-treated cells (**Figure 5D**), collectively suggesting that urothelial cells acquire immune function when exposed to an inflammatory environment.

### 3.5 Interferon gamma receptor 1 may be involved in TNFα-mediated effects on RT4 urothelial cells

**Figure 6:**
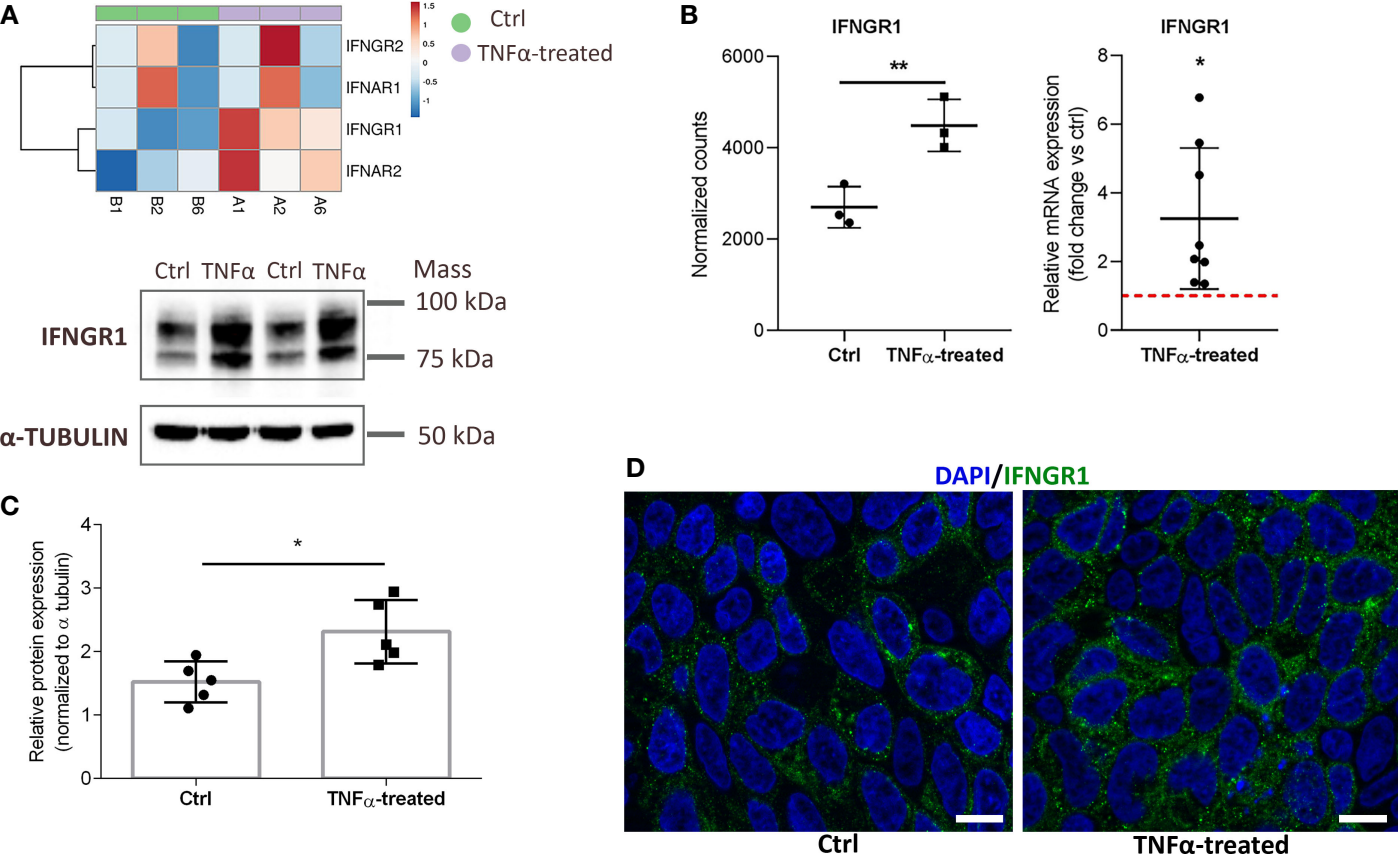
Upregulated mRNA and protein expression of IFNGR1 in TNFα-treated RT4 urothelial cells compared to controls. **(A)** Heatmap showing upregulated mRNA expression (RNA seq) of type I and II IFN receptors in TNFα-treated urothelial cells (purple, n=3 independent experiments: A1, A2, A6) and ctrl cells (green, n=3 independent experiments: B1, B2, B6). Rows are clustered using Euclidean distance and average linkage. Lower expression is indicated with blue, higher expression is indicated with red. **(B)** mRNA expression of IFNGR1 plotted as normalized RNA seq read counts (left) and mean ± SD fold change expression of an independent qPCR confirmatory analysis (right) in ctrl and TNFα-treated cells. Data are presented as mean ± SD normalized counts of three independent experiments (RNA seq) and as mean ± SD fold change expression in TNFα treated cells vs untreated (ctrl) cells (set to 1; dotted red line), determined in 8 independent experiments. **(C)** Confirmatory Western blot analysis showing significantly higher protein levels of IFNGR1 in TNFα-treated cells compared to controls. Shown are representative blots of 5 independent experiments, and mean ± SD of relative protein expression normalized to α-tubulin. **(D)** Representative images of three independent experiments of ctrl and TNFα-treated cells showing IFNGR1 (green) and DAPI (blue). Z-stack images were taken at the 63 x magnification at the same starting position and with the same distance (0.24 μm) between optical sections in ctrl and TNFα-treated samples. Scale bars: 10 µm. *p<0.05; **p<0.01.

Our data showed that TNFα not only alters the expression of key inflammatory cytokines such as IL1α, IL1β, and IL8 but also alters the expression of cytokine receptors such as type I and II interferon (IFN) receptors (IFNAR1, 2 and IFNGR1, 2) in RT4 cells (**Figure 6A**). The upregulated mRNA and protein expression of IFNGR1 was subsequently validated by qPCR (**Figure 6B**), Western blot (**Figure 6C**), and immunofluorescence (**Figure 6D**). IFNGR1 activates the downstream Janus kinase (JAK) signal transducer of transcription (STAT) signaling pathway (33), which is also significantly enriched in our TNFα-upregulated network (in addition to *IFNGR1, IL23A, CSF2, IL7R*, and I*L20RB*) (**Supplementary Table S2**), indicating the potential of JAK-STAT signaling pathway as a therapeutic target in IC/BPS.

### 3.6 Normal urothelial cells show similar phenotypic alterations as RT4 cancer urothelial cells in an inflammatory environment

**Figure 7:**
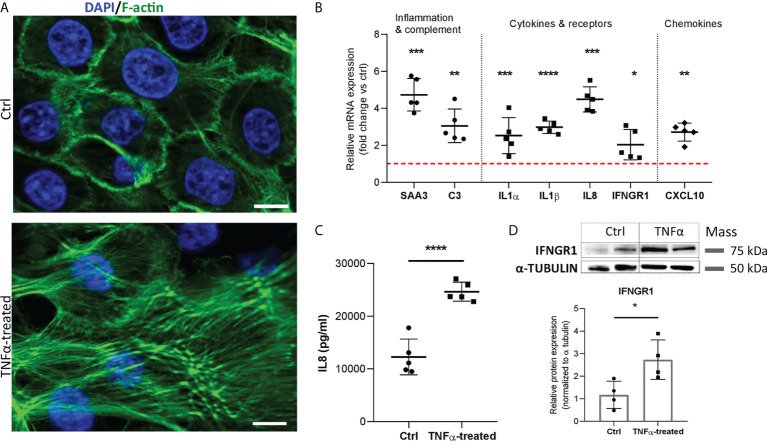
Morphologic, transcriptional and protein alterations in TNFα-stimulated normal porcine urothelial cells. **(A)** Representative images of 3 independent experiments showing F-actin (green) and DAPI (blue) of ctrl and TNFα-treated cells. Z-stack images were taken at the 63 x magnification at the same starting position and with the same distance (0.24 μm) between optical sections in ctrl and TNFα-treated samples. Scale bars: 10 µm. **(B)** Upregulated mRNA expression of genes involved in inflammatory response and complement cascade, proinflammatory cytokines, cytokine receptors, and chemokine in TNFα-treated cells, as compared to controls. Shown is mean ± SD fold change expression of target genes in TNFα-treated cells vs untreated cells (ctrl; set to 1; red dotted line), determined in 5 biological replicates by qPCR. **(C)** Protein levels of IL8 in the supernatants of TNFα-treated cells and ctrl (n=5 biological replicates). Shown are mean ± SD protein levels for each group. **(D)** Western blot analysis showing significantly higher protein expression of IFNGR1 in TNFα-treated cells compared to controls. Shown are representative blots of 4 biological replicates, and mean ± SD of relative protein expression normalized to α-tubulin. *p<0.05; **p<0.01; ***p<0.001; ****p<0.0001.

Although cancer urothelial cells represent a commonly cited *in vitro* model of IC/BPS, they may not accurately reflect IC/BPS pathology due to phenotype changes caused by the tumor microenvironment (25, 34). We therefore sought to also confirm our results by stimulating NPU cells. Based on viability assay and IL8 protein levels, 20 ng/ml TNFα and 48 h incubation of NPU cells were selected as optimal (**Supplementary Figure 2**). Our results show that TNFα-stimulated NPU cells exhibit similar changes in their phenotype as TNFα-stimulated RT4 cancer urothelial cells. We confirmed enhanced formation of long and thick actin stress fibers (**Figure 7A**), upregulated mRNA expression of the acute phase protein *SAA3*, complement component *C3*, chemokine *CXCL10*, several proinflammatory cytokines (*IL1α, IL1β, IL8*), and the cytokine receptor *IFNGR1* (**Figure 7B**). In addition, protein levels of IL8 (**Figure 7C**) and IFNGR1 (**Figure 7D**) were also significantly higher in TNFα-treated NPU cells compared with controls, as determined by ELISA and Western blot, respectively.

## 4 Discussion

Several studies exploiting the etiology and pathophysiology of IC/BPS suggest that urothelial cells may play a crucial role in the development of this disease (35–38). However, the precise transcriptional profile and function of these cells in an inflammatory environment (as in IC/BPS) were largely unclear. Here, we applied RNA seq technology to an established *in vitro* model of IC/BPS consisting of TNFα-treated cancer RT4 urothelial cells to characterize the overall effect of an inflammatory environment on their phenotype with genome-wide resolution. Our main findings were confirmed using a second *in vitro* IC/BPS model consisting of TNFα-treated NPU cells, contributing significantly to the body of knowledge on urothelial phenotype and function in bladder immune response.

The two most frequently cited *in vitro* IC/BPS models include LPS‐ and TNFα-induced inflammation of cancer RT4 and T24 urothelial cells (39). While LPS-induced model of inflammation is less appropriate since exclusion of bacterial infection is one of the criteria for the diagnosis of IC/BPS (1), TNFα, on the other hand, has been recognized as one of the most important proinflammatory cytokines involved in the pathophysiological process of IC/BPS (40, 41) and was therefore used as an inflammatory trigger in the current study.

Our transcriptome analysis revealed that the TNFα-upregulated transcriptional network in RT4 urothelial cells showed specific enrichment in several processes and pathways of innate immunity, such as granulocyte migration and chemotaxis, inflammatory response, and complement activation, as well as TLR-, NOD-like receptor- and NFkB-signaling pathways, suggesting an active role of urothelial cells in shaping tissue immunity in the urinary bladder. These specialized epithelial cells, equipped with pattern recognition receptors (PRRs), have previously been shown to provide early recognition of pathogens and elicit a rapid and robust proinflammatory immune response to combat urinary tract infections (42) also in our unpublished data on biomimetic urothelial *in vitro* model. However, their function in bladder immune response in IC/BPS is less known. IC/BPS patients have altered urothelial cell expression of proinflammatory cytokines and chemokines, including IL1β, and TNFα (14, 43), while urine samples from IC/BPS patients contain elevated levels of IL1β, IL8, CXCL1, CXCL10 (44–47), possibly derived from urothelial cells, as observed in our study, where significantly higher concentration of these cytokines and chemokines was determined in TNFα-stimulated cells. Abnormalities in specific proinflammatory signaling networks that contribute to alterations in bladder immune and structural cell phenotypes were recently uncovered by Su et al. (40), who performed single cell RNA seq in bladder tissues of patients with IC/BPS. They have also shown that immune response during IC/BPS is characterized by extensive infiltration of TNFα-producing neutrophil granulocytes and macrophages in bladder tissue (40). Their observations go hand in hand with our findings suggesting an active role of urothelial cells in shaping the bladder immunity through altered proinflammatory cytokine and chemokine signaling pathways. Further studies are needed to explore the involvement of urothelial cells in neutrophil and macrophage chemotaxis and migration, as well as the urothelial-immune cell communication, which may provide new therapeutic targets.

The TNFα-induced inflammatory *in vitro* model recapitulates key observations found in the urinary bladders of individuals with IC/BPS, as shown in the present study. Several enriched pathways and processes, observed in the bladder of patients with IC/BPS, especially those with Hunner’s lesions, including complement and coagulation cascades, cytokine-cytokine receptor interaction, chemokine signaling, granulocyte chemotaxis and migration, inflammatory response, TLR-, NOD-like receptor-, NFkB-and TNF-signaling pathways (14, 17, 32, 48, 49) were also identified in our study. These data support the utility of TNFα-treated RT4 urothelial cells as a suitable *in vitro* model for understanding IC/BPS mechanisms and confirm the role of TNFα signaling as an important component in the associated pathology.

The present study also identifies novel upregulated gene targets of TNFα in urothelial cells, such as the acute phase reactant SAA, complement component C3, and the cytokine receptor IFNGR1. It should be noted here that we and others have used RT4 or T24 cancer cell lines to model bladder urothelium *in vitro* (18–21). However, these cells may not accurately reflect the *in vivo* situation due to alterations in phenotype caused by the cancerous transformation. Hence, we also confirmed our new findings by stimulating bladder normal urothelial cells. As normal human urothelial cells are difficult to obtain for ethical reasons, we used normal porcine urothelial cells (NPU), which are available in sufficient amounts and have many structural, molecular and physiological properties that correspond to normal human urothelial cells (27, 50–53). We discovered that TNFα-stimulated NPU cells show similar changes in mRNA and protein expressions as TNFα-stimulated RT4 urothelial cells pointing towards a high degree of interspecies similarities suggesting that both cell types can be used as *in vitro* models to mimic IC/BPS. Based on our observations, we suggest that SAA, C3 and IFNGR1 should be further explored as potential therapeutic targets of IC/BPS.

SAA represents a family of highly homologous acute-phase proteins that have been remarkably conserved in vertebrate evolution (54). Levels of SAA increase substantially in response to trauma, infection, inflammation, and neoplasia and serve to regulate lipid metabolism and transport, immune cell chemotaxis, and other inflammatory processes (55). In humans, SAA1 and SAA2 are considered acute phase reactants, whereas in pigs, the SAA3 isoform is the major acute phase protein induced in response to an inflammatory stimulus (56), which was also shown in the present study. TNFα is one of the major inducers of SAA synthesis in the liver, and it has also been shown that the production of SAA is increased in intestinal epithelial cells following TNFα stimulation, possibly contributing to the development of inflammatory bowel disease (57, 58). In urothelial cells, expression of SAA has been shown to increase upon infection with uropathogenic bacteria *E.coli* (59), whereas Lannergard et al. proposed that it might serve as a sensitive systemic biomarker of uncomplicated cystitis due to urinary tract infections (UTI) (60). While SAA plays a protective role in UTI, its continued production in chronic inflammation may promote the development and progression of IC/BPS. Based on the following, we suggest that SAA might be a useful diagnostic marker of IC/BPS, which should be validated in future clinical studies.

C3 is an important component of the complement cascade and plays a critical role in the clearance of invading pathogens. It is synthesized and produced primarily by hepatocytes (61), but also by other cell types, including epithelial cells (62). While complement components play an important role in regulating local immune responses, their uncontrolled and sustained production can trigger severe inflammatory processes and lead to tissue damage (63). C3 is produced and secreted in large amounts by intestinal epithelial cells after TNFα stimulation (64, 65), but little is known about its production and secretion by urothelial cells. Overexpression of C3 has been shown to reduce the expression of E-cadherin (66), a key component of adherent junctions essential for cell adhesion and maintenance of the urothelial permeability barrier. A number of studies have shown that E-cadherin expression is decreased in the urothelium of IC/BPS patients (10, 35, 36, 67, 68), which is also suggested by our *in vitro* models. Specifically, we showed TNFα-induced stress fiber formation pointing towards actin remodeling and cytoskeletal rearrangement that may increase the permeability of urothelial cells and thus induce barrier dysfunction, as already shown for TNFα-stimulated endothelial cells (30, 31). C3 might therefore be indirectly linked to urothelial permeability dysfunction in IC/BPS, which should be further validated.

IFNGR1, along with type I IFN receptors, which were also upregulated in our study, may be of particular interest in IC/BPS since they are considered to be pivotal players in various inflammatory and autoimmune diseases (69, 70). Activation of IFN receptors triggers stimulation of a JAK/STAT pathway, which was also significantly enriched in our study. The IFNGR1-JAK1 activation pathway has recently been shown to have an impact on the survival and differentiation of urothelial cells, as well as affects their interaction with immune cells (71). It appears that not only does the leaky urothelium trigger an inflammatory response and a downstream cascade of evens, but also inflammatory mediators released by urothelial cells affect their differentiation and compromise the permeability barrier. Thus, whether inflammation is the cause or the consequence of a disrupted permeability barrier in IC/BPS remains to be elucidated.

Our study has its limitations; first, the TNFα concentrations used were relatively high (20 ng/ml) and inflammation was induced over a short time period of 24 h. It should be noted here that the concentration of TNFα, used in the current (20 ng/ml) and previously published *in vitro* studies is significantly higher (approximately five to ten thousand times) compared to the concentration of TNFα measured in urine (approximately 2-4 pg/ml) of patients with IC/BPS (72). The concentration of TNFα used for the *in vitro* model described in the current study has been selected based on viability assay and the ability to elicit a prominent inflammatory and immune response in urothelial cells that could be determined with the methods used (RNA seq, qPCR, WB, ELISA) and reached statistical significance vs untreated cells. When lower concentrations of TNFα were used (for example 2 ng/ml), only a slight increase in secreted IL8 levels was detected compared to untreated cells, so higher TNFα concentrations were necessary in order to be able to describe this particular *in vitro* model and compare our findings with the findings determined in the bladders of patients with IC/BPS. Although the *in vitro* model described here recapitulated the key findings from the transcriptomes of bladders from patients with IC/BPS, especially those with Hunner’s lesions in whom the inflammatory component is more prominent, caution is still required when interpreting these results due to the high TNFα concentrations used. A lower concentration of TNFα, used repeatedly over a longer period of time might better mimic the low-grade inflammatory environment observed in IC/BPS. Future studies should examine whether urothelial cells can elicit a prominent inflammatory response when stimulated with lower, clinically relevant concentrations of TNFα, using more sensitive methods for detection, such as single cell RNA seq, and 3D *in vitro* models that better mimic the structure of the bladder and account also for interactions between different cell types. Urothelial cells in such models might be much more sensitive also to lower concentrations of TNFα, such as the ones measured in urine samples of IC/BPS patients. Second, RT4 cell line, isolated from non-invasive grade I transitional cell carcinoma and NPU cells used were not terminally differentiated and thus may not fully reflect the characteristics and functionality of normal bladder urothelial cells (29, 52, 73). Although such cells are absent or present only in limited numbers in the bladders of IC/BPS patients due to urothelial denudation (74). Third, the *in vitro* model utilized here consisted only of urothelial cells grown until reaching 80-90% confluency and therefore still in monolayers which misses the importance of multiple cell-cell interactions, found *in vivo*. Further clinical studies, animal studies or studies using a 3D *in vitro* model containing various bladder structural and immune cell types are needed to validate our results. On the contrary, using only one particular cell type allowed us to decipher in depth their transcriptome by RNA seq technology which cannot be determined using whole bladder tissue of animal models or human patients. Namely, bladder tissue samples consist of several cell populations and bulk RNA seq technology used here enables only the analysis of average global gene expression of all cell types included in the sample (75). Our study therefore represents an important and informative resource database for future studies exploring specifically the phenotype and function of urothelial cells, not only in IC/BPS, but also in other bladder diseases, such as infectious cystitis and bladder cancer. Our observations have important implications in enhancing our knowledge of the bladder tissue-specific immunity and in identifying novel mechanisms in the urothelium as potential targets for future pharmacologic intervention in IC/BPS.

## Data availability statement

The datasets presented in this study can be found in online NCBI repository (76) (https://www.ncbi.nlm.nih.gov/) with accession number GSE202576.

## Author contributions

TK, MEK, and PV designed the experiments. TK and DP performed the experiments. TK performed the statistical analyses and visualized the data. MEK, AE and PV provided critical input to data visualization and interpretation. TK wrote the original draft. All authors reviewed and edited the final draft. All authors have seen and approve the manuscript and its contents, as well as are aware of the responsibilities connected with the authorship.

## Funding

This work was supported by the Slovenian Research Agency (J3-2521, J7-2594 and P3-0108) and MRIC UL IP-0510 Infrastructure program.

## Acknowledgments

The authors would like to acknowledge the support of Nada Pavlica Dubarič in Western blot experiments, Sanja Čabraja in cell culture experiments and Nina Visočnik in performing ELISA assays.

## Conflict of interest

The authors declare that the research was conducted in the absence of any commercial or financial relationships that could be construed as a potential conflict of interest.

## Publisher’s note

All claims expressed in this article are solely those of the authors and do not necessarily represent those of their affiliated organizations, or those of the publisher, the editors and the reviewers. Any product that may be evaluated in this article, or claim that may be made by its manufacturer, is not guaranteed or endorsed by the publisher.

## Supplementary material

Supplementary Figure 1 Optimization of TNFα concentration and time of incubation in RT4 urothelial cells based on viability assay and protein levels of IL8. **(A)** Viability of RT4 urothelial cells stimulated with increasing concentrations of TNFα (0, 2, 10, 20, 50, 100 ng/ml) in serum-free basal media for 24 h. Shown are mean ± SD values measured in triplicates in three independent experiment, expressed as percentage of luminescence signal intensity of untreated controls (set to 100). **(B)** Protein levels of IL8 released in the supernatants of RT4 cells treated with increasing concentrations of TNFα (0, 2, 10, 20, 50 ng/ml). **(C)** Protein levels of IL8 released in the supernatants of RT4 cells treated with 20 ng/ml TNFα for 24, 48 or 72 h. Shown are mean ± SD values measured in three independent experiments.

Supplementary Figure 2 Optimization of TNFα concentration and time of incubation in normal porcine urothelial cells based on viability assay and protein levels of IL8. **(A)** Viability of NPU cells stimulated with increasing concentrations of TNFα (0, 2, 10, 20, 50, 100 ng/ml) in serum-free basal media for 48 h. Shown are mean ± SD values measured in triplicates in three biological replicates, expressed as percentage of luminescence signal intensity of untreated controls (set to 100). **(B)** Protein levels of IL8 released in the supernatants of NPU cells treated with increasing concentrations of TNFα (0, 2, 10, 20, 50 ng/ml). Shown are mean ± SD values measured in triplicates in two biological replicates. **(C)** Protein levels of IL8 released in the supernatants of NPU cells treated with 20 ng/ml TNFα for 24 or 48 h.
