# Supplementary figures and images for: Comprehensive transcriptome profiling of urothelial cells following TNFα stimulation in an in vitro interstitial cystitis/bladder pain syndrome model
Source: Front Immunol. 2022 Aug 15;13:960667. doi: 10.3389/fimmu.2022.960667 (PMC9421144; doi:10.3389/fimmu.2022.960667)

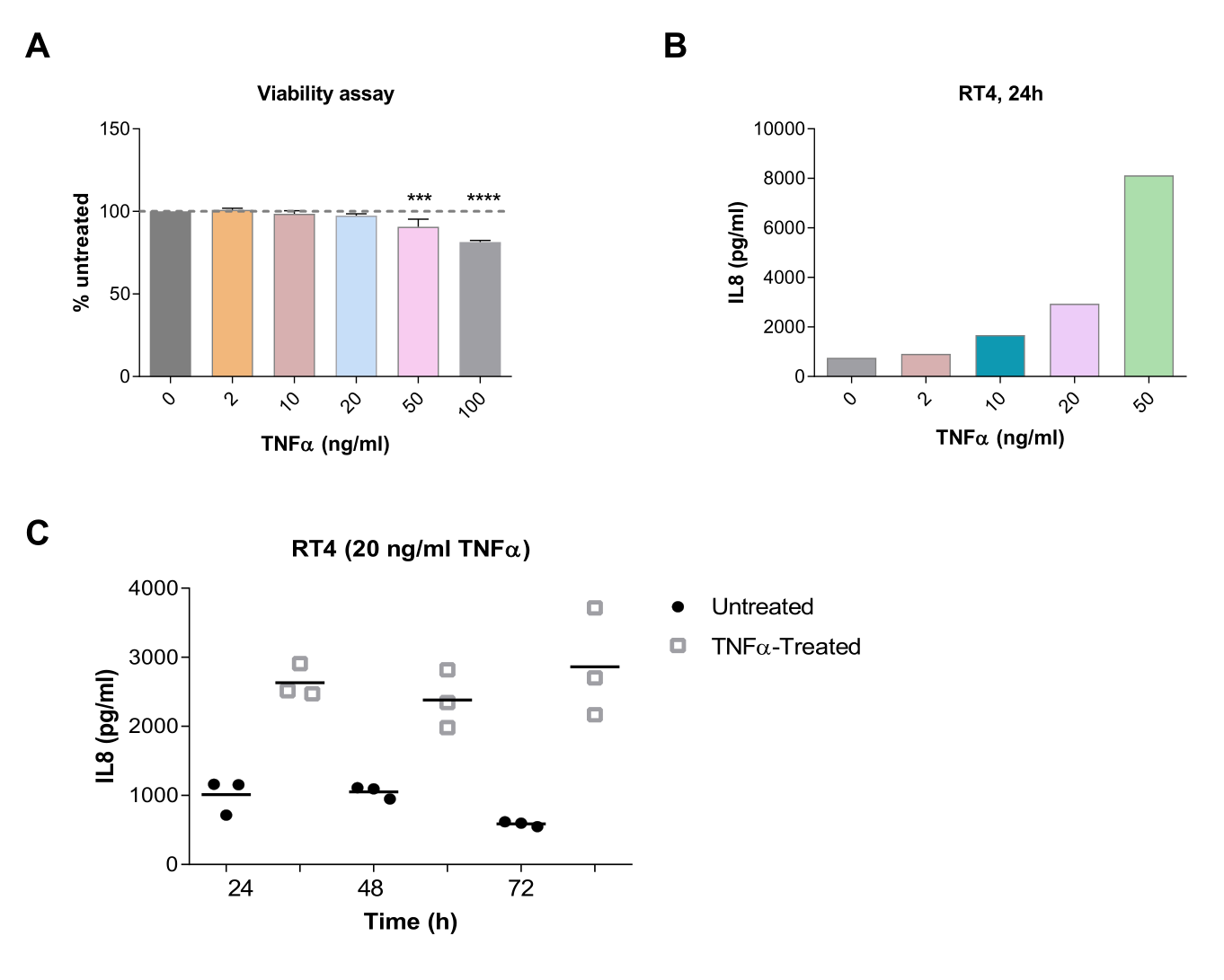

Supplement: Supplementary Figure 1 — Optimization of TNFα concentration and time of incubation in RT4 urothelial cells based on viability assay and protein levels of IL8. (A) Viability of RT4 urothelial cells stimulated with increasing concentrations of TNFα (0, 2, 10, 20, 50, 100 ng/ml) in serum-free basal media for 24 h. Shown are mean ± SD values measured in triplicates in three independent experiment, expressed as percentage of luminescence signal intensity of untreated controls (set to 100). (B) Protein levels of IL8 released in the supernatants of RT4 cells treated with increasing concentrations of TNFα (0, 2, 10, 20, 50 ng/ml). (C) Protein levels of IL8 released in the supernatants of RT4 cells treated with 20 ng/ml TNFα for 24, 48 or 72 h. Shown are mean ± SD values measured in three independent experiments. [file Image_1.tiff]

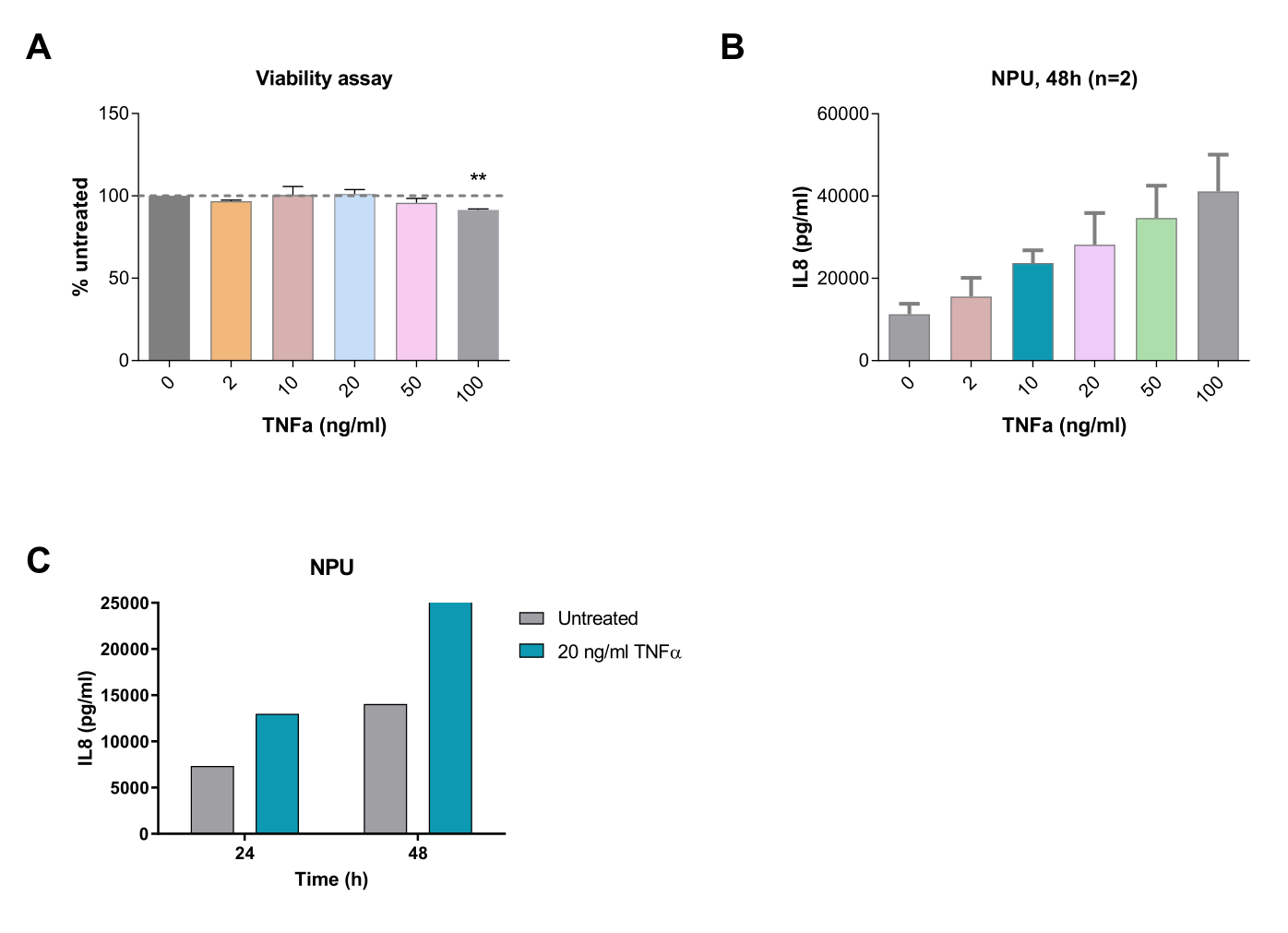

Supplement: Supplementary Figure 2 — Optimization of TNFα concentration and time of incubation in normal porcine urothelial cells based on viability assay and protein levels of IL8. (A) Viability of NPU cells stimulated with increasing concentrations of TNFα (0, 2, 10, 20, 50, 100 ng/ml) in serum-free basal media for 48 h. Shown are mean ± SD values measured in triplicates in three biological replicates, expressed as percentage of luminescence signal intensity of untreated controls (set to 100). (B) Protein levels of IL8 released in the supernatants of NPU cells treated with increasing concentrations of TNFα (0, 2, 10, 20, 50 ng/ml). Shown are mean ± SD values measured in triplicates in two biological replicates. (C) Protein levels of IL8 released in the supernatants of NPU cells treated with 20 ng/ml TNFα for 24 or 48 h. [file Image_2.tiff]
