# Supplementary material for: Comprehensive transcriptome profiling of urothelial cells following TNFα stimulation in an in vitro interstitial cystitis/bladder pain syndrome model
Source: Front Immunol. 2022 Aug 15;13:960667. doi: 10.3389/fimmu.2022.960667 (PMC9421144; doi:10.3389/fimmu.2022.960667)
Supplement: Supplementary file 3 [file Table_1.docx]

| **Human primers** | | |
| --- | --- | --- |
| **mRNA** | **Forward sequence (5'-3')** | **Reverse sequence (5'-3')** |
| GAPDH | CGGATTTGGTCGTATTGG | TCCTGGAAGATGGTGATG |
| IL1α | AGATGCCTGAGATACCCAAAACC | CCAAGCACACCCAGTAGTCT |
| IL1β | TTCGACACATGGGATAACGAGG | TTTTTGCTGTGAGTCCCGGAG |
| IL8 | TTTTGCCAAGGAGTGCTAAAGA | AACCCTCTGCACCCAGTTTTC |
| IL23 | CTCAGGGACAACAGTCAGTTC | ACAGGGCTATCAGGGAGCA |
| IL32 | TGGCGGCTTATTATGAGGAGC | CTCGGCACCGTAATCCATCTC |
| CXCL1 | AACCGAAGTCATAGCCACACT | TCTGGTCAGTTGGATTTGTCACT |
| CXCL3 | CGCCCAAACCGAAGTCATAG | GCTCCCCTTGTTCAGTATCTTTT |
| CXCL5 | AGCTGCGTTGCGTTTGTTTAC | TGGCGAACACTTGCAGATTAC |
| CXCL10 | AGTGGCATTCAAGGAGTACCT | GATCTCAACACGTGGACAAAATTG |
| CXCL11 | GACGCTGTCTTTGCATAGGC | GGATTTAGGCATCGTTGTCCTTT |
| IFNGR1 | AGCAGGAAGTCGATTATGATCCC | CTGGCACTGAATCTCGTCACA |
| SAA1 | AGAGATTCTTTGGCCATGGTG | TCGGAAGTGATTGGGGTCTT |
| SAA2 | CTGCTCCTTGGTCCTGAGTG | ACTGATCACTTCTGCAGCCC |
| C3 | GGGGAGTCCCATGTACTCTATC | GGAAGTCGTGGACAGTAACAG |
| LTB | GACGAAGGAACAGGCGTTTCT | GTAGCCGACGAGACAGTAGAG |
| TNFRSF1B | TGAAACATCAGACGTGGTGTG | TGCAAATATCCGTGGATGAAGTC |
| TNFRSF14 | ACCGAGAGTCAGGACACCC | AGCAAACAATGACGATGACGA |
| TNFAIP3 | TTGTCCTCAGTTTCGGGAGAT | ACTTCTCGACACCAGTTGAGTT |
| BIRC3 | TTTCCGTGGCTCTTATTCAAACT | GCACAGTGGTAGGAACTTCTCAT |
| **Porcine primers** | | |
| ACTB | CGAGACCTTCAACACCCCAG | AGTCCATCACGATGCCAGTG |
| IL1α | AGAAGAAATCATCAAGCCCAGATC | ATTGCGGGCATCATTCAGGATG |
| IL1β | GGGCTTTTGTTCTGCTTGAG | CCAAAGAGGGACATGGAGAA |
| IL8 | TTGTGTTGGCATCTTTACTGAGA | AAGAGAACTGAGAAGCAACAACA |
| CXCL10 | ACTCAAGGAATACCTCTCTCCAG | AAGGACCTCGGATTAACAGGT |
| IFNGR1 | AAACGGAAGCGAGACCACAG | GTTCAGCGAGGACACTGGAA |
| SAA3 | CTCAAGGAAGCTGGTCAAGG | GGACATTCTCTCTGGCATCG |
| C3 | GTCACCATTGAGACACCTGAAG | CATGTTGACCAGCTCTGGGA |

**Supplementary Table S1. List of mRNA primers used for qPCR.**
